# Updates from a single-center phase 2 study of PD-1 inhibitor combined with hypomethylating agent plus CAG regimen in patients with relapsed/refractory acute myeloid leukemia

**DOI:** 10.3389/fimmu.2025.1533467

**Published:** 2025-04-17

**Authors:** Hui-Sheng Zhou, Yong-Feng Su, Jun Wang, Ya-Lei Hu, An Wang, Lei Xu, Yi-Zhi Wang, Xuan Zheng, Yu-Qing Li, Kai-Li Min, Chun-Ji Gao, Dai-Hong Liu, Xiao-Ning Gao

**Affiliations:** ^1^ Senior Department of Hematology, The Fifth Medical Center of Chinese PLA General Hospital, Beijing, China; ^2^ Graduate School, Chinese PLA General Hospital, Beijing, China; ^3^ State Key Laboratory of Experimental Hematology, Senior Department of Hematology, The Fifth Medical Center of Chinese PLA General Hospital, Beijing, China

**Keywords:** acute myeloid leukemia, DNA hypomethylation agent, PD-1 inhibitor, relapse, refractory

## Abstract

**Introduction:**

Anti-PD-1 monotherapy has shown limited clinical efficacy in patients with relapsed/refractory acute myeloid leukemia (r/r AML). Our study aimed to analyze the effectiveness and safety of combining tislelizumab with a hypomethylating agent (HMA) plus CAG regimen in treating patients with r/r AML, with an increased sample size and in comparison, with a historical control group for more reliable data support (ClinicalTrials.gov identifier NCT04541277).

**Methods:**

The study included a total of 37 patients with r/r AML who received the tislelizumab + HMA + CAG regimen.

**Results:**

The overall response rate was 69.4%, with a median overall survival of 12.1 months and event-free survival of 6.2 months. Multivariate analysis revealed that patients aged 40 or above exhibited a higher response rate, while those with lower leukemia burden (bone marrow blast percentage <40%) demonstrated improved overall survival and event-free survival. Additionally, bridging allogeneic hematopoietic stem cell transplantation was associated with extended event-free survival. Grade 2-3 immune-related adverse events were observed in 8.5% of patients, and no deaths were directly attributed to these events. After propensity score matching, the inclusion of tislelizumab appeared to positively influence the overall response rate and event-free survival compared to historical controls treated with HMA + CAG regimen.

**Discussion:**

Overall, the combination regimen improved response rates while maintaining low incidence and severity of immune-related adverse events for r/r AML patients.

**Clinical trial registration:**

https://clinicaltrials.gov/, identifier NCT04541277.

## 1 Introduction

Acute myeloid leukemia (AML) is a complex and heterogeneous hematological malignancy characterized by the rapid proliferation of abnormal myeloid cells in the bone marrow (BM). The prognosis for patients with AML remains challenging, particularly due to the high rate of relapse following initial treatment. More than 50% of patients with AML will experience a relapse within 3 years after their initial diagnosis (1, 2), indicating that current therapeutic strategies may not be sufficiently effective in achieving long-term remission. This is even more critical for those who experience an initial relapse, as their 1-year survival rate is only 29% (3). In addition, salvage therapies, which are given after the failure of standard treatments, show a response rate of only 21% in patients with relapsed or refractory (r/r) AML (4). The median overall survival (OS) for patients with r/r AML receiving conventional salvage therapy is only 3.3 months, and <10% of patients with r/r AML achieve 5-year OS rates (4). Therefore, the high incidence of r/r AML and the unsatisfactory efficacy of current treatments necessitate the development of new treatment regimens to improve response rates and prolong survival.

Programmed cell death 1 (PD-1) and programmed cell death ligand (PD-L1) are critical immune checkpoint molecules involved in maintaining immune tolerance, but tumor cells exploit this pathway to evade detection by the immune system (5). Consequently, blocking the PD-1/PD-L1 interaction can enhance the anti-tumor activity of T cells, making PD-1 inhibitors promising for treating certain solid tumors. Research has shown that targeting the PD-1/PD-L1 pathway with inhibitors has led to significant improvements in patient outcomes for various types of cancer. This has sparked interest in exploring the potential benefits of using PD-1 inhibitors in AML treatment. However, there is limited literature on the use of PD-1 inhibitors in AML.

Research using mouse models of AML have demonstrated that tumor progression correlates with an increase in regulatory T cells (Tregs) among infiltrating CD8^+^ T cells in the liver, resulting in upregulation of PD-1 expression and subsequent impairment of CD8^+^ T cell functionality (6, 7). Inhibition of the PD-1/PD-L1 interaction facilitated the depletion of Tregs and enhanced cluster of differentiation 8 (CD8^+^) T cell activity. This study elucidates the mechanism by which PD-1/PD-L1 interactions inhibit T-cell function and promote immune evasion in leukemia. Related clinical studies have demonstrated that the frequency of relapse in patients with AML correlates positively with the proportion of T cells expressing PD-1 in BM (8). Furthermore, patients with AML exhibiting elevated levels of PD-1, PD-L1, or PD-L2 expression experienced a significantly lower OS compared with those showing reduced expression (9). These findings substantiate the association between high levels of PD-1/PD-L1 at immune checkpoints and poor prognosis in AML, indicating that targeting the PD-1/PD-L1 signaling pathway may represent a promising therapeutic strategy for this malignancy.

Although researchers have attempted to use PD-1 inhibitor monotherapy for the treatment of AML, its efficacy is limited. Among the 8 patients with AML, that were assessed, only 1 patient exhibited a response to CT-011, a humanized antibody interacting with PD-1, demonstrating a reduction in peripheral blood blasts from 50% to 5% and remaining free from platelet transfusions for 9 months (10). Hypomethylating agents (HMAs) upregulate a wide variety of genes, including previously silenced leukemia tumor-associated antigens and neoantigens, serving as targets for AML-specific immune responses (11). Conversely, HMAs also induce the expression of PD-1/PD-L1 (12). This underscores the potential utility of PD-1 inhibitors in overcoming tumor immune resistance. The aforementioned insights indicate that the combination of PD-1 antibodies and HMAs could achieve enhanced therapeutic antitumor efficacy than anti-PD-1 monotherapy. A study investigating the combination regimen of azacitidine and nivolumab in patients with r/r AML reported an overall response rate (ORR) of 33%, compared with historical controls receiving azacitidine alone, that demonstrated a response rate of 20% (13). In treatment-naïve patients, the ORR reached as high as 52%. The interim results of a study investigating the combination of nivolumab and high-dose chemotherapy for the treatment of AML showed that among 30 newly diagnosed AML patients, the CR/complete remission with incomplete blood count recovery (CRi) rate was 73%, and the mortality rate at 8 weeks was 6% (14). Based on these findings, we speculate that the combination of anti-PD-1 antibodies with conventional chemotherapy and HMAs may overcome drug resistance, enhance anti-tumor activity, and thereby further improve the therapeutic efficacy in treating r/r AML. We have initiated a phase 2 trial investigating the efficacy and safety of tislelizumab combined with decitabine/azacytidine plus cytarabine, aclarubicin/idarubicin, and G-CSF (CAG) regimen in treating patients with AML who failed to the previous chemotherapy (NCT04541277). Our preliminary results show that this combination therapy is an effective and safe treatment (15). This study is an extension of our earlier work. We have increased the sample size, extended the follow-up period, and incorporated new analytical methods, including propensity score matching (PSM) and comparative evaluation, to further analyze the clinical efficacy and safety of the tislelizumab + HMA + CAG regimen in the treatment of patients with r/r AML, while exploring the impact of clinical and molecular biological characteristics on the clinical response and patient survival.

## 2 Methods

### 2.1 Study design and treatment

This was an open-label nonrandomized single-center phase 2 study of tislelizumab + HMA + CAG in high-risk AML or AML patients older than 60 years of age who are unfit for standard-dose chemotherapy (NCT04541277). The primary endpoint of the study was ORR with the best response achieved. The criteria for efficacy refer to the AML European Leukemia Network (ELN) guidelines (16). ORR is defined as the proportion of patients who achieve either a CR or CRi or partial response (PR) to therapy. Responders were defined as those who achieved any of these responses, and non-responders were defined as those who did not achieve CR, CRi, or PR. Adverse events (AEs) were graded using the Common Terminology Criteria for Adverse Events (CTCAE) version 5.0. Toxicity is defined as any clinically obvious grade 3 or 4 nonhematologic AEs or death caused by the study drugs. Secondary endpoints include the percentage of subjects with MRD-negative CR or CRi, OS, event-free survival (EFS), and duration of response (DOR), and incidence of adverse events (AEs). The MRD negative status was defined as leukemia immunophenotyping negative by multiparameter flow cytometry whereas MRD positive was defined as naive myeloid cells with abnormal immunophenotype ≥ 0.01%. OS is measured from the start of treatment to the date of death from any cause or reviewed at the date of the last follow-up. The EFS is measured from the start of treatment until treatment fails, relapses, or death occurs due to any cause (calculated as the cause that occurred first). Among responders, DOR was defined as the time interval between the response date and the recurrence of disease or death from any cause, whichever occurred first. The study was approved by the Ethics Committee of the Chinese PLA General Hospital (No. S2020-296-01) and conducted in accordance with the Declaration of Helsinki and the International Conference on Harmonization Good Clinical Practice guidelines. Written informed consent was obtained from all participants before treatment.

The treatment regimen consisted of a daily intravenous decitabine 20 mg/m^2^, days 1-7 or azacitidine 75 mg/m^2^ subcutaneously daily, days 1-5; cytarabine 100 mg intravenously every 12 hours, days 1-5; idarubicin 10 mg intravenously day 1, 3, and 5 or a daily intravenous aclarubicin 20 mg, days 1-5; G-CSF 5 μg/kg/day subcutaneously from day 0 to the end of chemotherapy when the white blood cell (WBC) count exceeds 10×10^9^/L; and tislelizumab 200 mg intravenously day 6 or day 8 (started the day after chemotherapy was stopped). The dose adjustment of cytarabine was in accordance with our previously report (15). Each cycle lasted for 28 days and was repeated every 4-6 weeks depending on the absence of disease progression and count recovery or unacceptable toxicity. During the treatment, the patients were given the best symptomatic support treatment such as anti-infective agents, stimulating hematopoietic and component blood transfusions.

Baseline assessments included a comprehensive medical history and physical examination, complete blood count, chemical profile, serum electrolytes, thyroid hormone and cortisol testing, BM aspiration, and pregnancy tests. Assessment of response to treatment was performed by examining BM aspiration at the end of each cycle.

### 2.2 Eligibility

The trial population consisted of patients with r/r AML who had an insufficient response to the first induction, with a <50% proportional reduction in blasts and the presence of >15% blasts, or a failure to achieve complete remission (CR) after 2 courses of induction (17), or developed relapse (16). Moreover, minimal residual disease (MRD)-positive patients were eligible to receive the study therapy. Patients met the following criteria: aged ≥18 years with an Eastern Cooperative Oncology Group (ECOG) performance score of ≤2, serum creatinine ≤1.5 × upper limit of normal range (ULN); serum bilirubin ≤1.5 × ULN, serum aspartate aminotransferase or alanine aminotransferase ≤2.5 × ULN. Patients with a history of inflammatory bowel disease, autoimmune disease, or any other severe and/or uncontrolled medical condition (e.g., uncontrolled diabetes; cardiovascular diseases, including congestive heart failure, myocardial infarction within 6 months, and active uncontrolled infection; chronic renal failure; or poorly controlled hypertension) and who were taking immune suppression medications or steroids (>10 mg/day) were excluded.

### 2.3 Statistical analysis

It was hypothesized that r/r AML patients would achieve a 60% ORR with the combination of tislelizumab + HMA + CAG regimen treatment. A Simon’s two-stage design tested the null hypothesis of a 35% ORR for historical controls. A minimum sample size of 27 patients was determined to achieve an 80% power for detecting a 25% difference at a one-sided alpha level of 0.05. Enrollment concluded following an interim futility analysis conducted in accordance with Simon’s stopping rules.

Descriptive statistics were used to describe continuous variables expressed in medians and ranges. Percentages and frequencies were used to describe categorical variables. The Cochran-Mantel-Haenszel -χ^2^ (CMH -χ^2^) test (the Fisher precision test for cell frequencies < 5) or the Wilcoxon rank sum test was used to compare the continuous variables between groups. To account for differences in variables affecting survival between cohorts, PSM was employed to select historical control groups using a 1:2 matching ratio with a tolerance level of 0.1 via the nearest neighbor method. The following variables were included: age (continuous), disease status (refractory or relapsed), and bone marrow blast percentage (continuous). To verify the stability of the results, we conducted a sensitivity analysis using multivariate analysis of prognostic factors for the ORR in patients with AML following PSM. The variables with a *P*-value <0.1 in univariate analysis were further analyzed by binary logistic regression model to identify the independently relevant factors of ORR. The survival analysis was performed using the Kaplan-Meier method of log-rank test with 95% confidence interval (CI). A multivariate analysis was performed for related variables with a *P*-value <0.1 in the univariate analysis, and time-dependent Cox regression models were used to identify the independently relevant factors of survival. A *P*-value <0.05 was considered statistically significant. All statistical analyses were performed using Stata 17.0 software (Stata Corporation, College Station, TX, USA) and SPSS 26.0 software (IBM Corporation, Armonk, NY, USA).

## 3 Results

### 3.1 Patient population

From September 15, 2020, to February 29, 2024, a total of 48 patients received the tislelizumab + HMA + CAG regimen treatment at the Chinese PLA General Hospital in Beijing. Among them, 37 patients met the inclusion criteria of r/r AML and received at least 1 cycle of treatment and were included in the current analysis with a data cutoff of July 1, 2024 (**Supplementary Figure 1**); of which 27 patients reported previously (15). Baseline demographic and clinical characteristics are presented in **Table 1**. Median age was 45 years (range, 18-71 years); 11 patients were women. Thirty-one patients had *de novo* AML and 6 patients had secondary AML (antecedent MDS, n=5; therapy-related, n=1). Four patients had extramedullary leukemia. Twenty-six patients (70.3%) had refractory disease, and 11 patients (29.7%) had disease relapse. Prior treatment included HMA + CAG regimen (n=8; 21.6%), standard “3 + 7” regimen (idarubicin/daunorubicin + cytarabine) (n=25; 67.6%), high/intermediate-dose cytarabine-based therapy (n=8; 21.6%). In addition, 3 patients had undergone allogeneic hematopoietic stem cell transplantation (allo-HSCT) prior to receiving tislelizumab + HMA + CAG regimen treatment.

**Table 1:** Baseline characteristics of patients with AML.

Variable	Number	Percent
Total number of patients	37	
Age, median, years (range)	45 (18 - 71)	
< 40	12	32.43
≥ 40	25	67.57
Gender		
Male	26	70.27
Female	11	29.72
Cycles of prior therapies		
1	21	56.8
2	7	18.9
≥3	9	27.3
Prior therapies^a^		
Idarubicin/daunorubicin + cytarabine	25	67.6
IDAC-based/HDAC-based	8	21.6
HMA + CAG	8	21.6
Antecedent allo-HSCT	3	8.1
Subsequent allo-HSCT	17	46.7
Diagnosis		
AML de novo	31	83.8
Secondary AML	6	16.2
Therapy-related	1	2.7
Progressed from MDS	5	13.5
With extramedullary leukemia	4	10.8
Disease status		
Refractory	26	70.3
Relapse	11	29.7
Bone marrow blast, median, % (range)	33 (1.6 - 83.6)	
White blood cell count, median, × 10^9^/L (range)	3.3 (0.6 - 80.8)	
Platelets, median, × 10^9^/L (range)	79 (2 - 310)	
Hemoglobin, median, g/L (range)	70.5 (37 - 140)	
ELN 2022 risk classification		
Favorable	14	37.8
Intermediate	9	24.4
Adverse	14	37.8
Cytogenetics		
Normal karyotype	20	54.1
t(8;21)(q22;q22)	7	18.9
inv(16)(p12;q22)	1	2.7
Monosomal karyotype	1	2.7
Complex karyotype	2	5.4
t(3;21)	1	2.7
Others	5	13.5
Genetic mutations		
CEBPA-bZip	7	18.9
RUNX1	7	18.9
WT1	6	16.5
FLT3-ITD	5	13.5
TET2	5	13.5
ASXL1	5	13.5
IDH2	4	10.8
TP53	4	10.8
U2AF1	4	10.8
FLT3-TKD	3	8.1
DNMT3a	3	8.1
PTPN11	3	8.1
ETV6	2	5.4
JAK2	2	5.4
JAK3	2	5.4
KIT	2	5.4
KDM6A	2	5.4
NRAS	2	5.4
KRAS	2	5.4
BCOR	2	5.4
GATA2	2	5.4
SETBP1	2	5.4
SRSF2	2	5.4
CALR	1	2.7
CSF3R	1	2.7
FBXW7	1	2.7
ITPKB	1	2.7
MED12	1	2.7
MPL	1	2.7
PHF6	1	2.7
NPM1	1	2.7
RAD21	1	2.7
SLC22A1	1	2.7
SLCO1A2	1	2.7
Fusion genes		
AML1-ETO	7	18.9
MLL-AF6	2	5.4
CBFβ-MYC11	1	2.7
TLS-ERG	1	2.7

a The number and percentage represent patients, not a percentage from total prior therapy.

allo-HSCT, allogeneic hematopoietic stem cell transplantation AML; acute myeloid leukemia; ASXL1, additional sex comb like 1; CEBPA-bZip, CCAAT/enhancer-binding protein α- basic leucine zipper; HMA, hypomethylating agent; CAG, decitabine/azacitidine, cytarabine, idarubicin/aclarubicin, G-CSF; ELN, European LeukemiaNet; FLT3-ITD, FMS-like tyrosine kinase-3 internal tandem duplication; HDAC, high-dose cytarabine–based; IDH2, isocitrate dehydrogenase 2; IDAC, intermediate-dose cytarabine-based; MDS, myelodysplastic syndromes; RUNX1, runt-related transcription factor 1; TP53, tumor protein p53; TET2, ten-eleven translocation 2; U2AF1, U2 small nuclear RNA auxiliary factor 1; WT1, wilms' tumor 1.

### 3.2 Treatment duration and response

**Figure 1:**
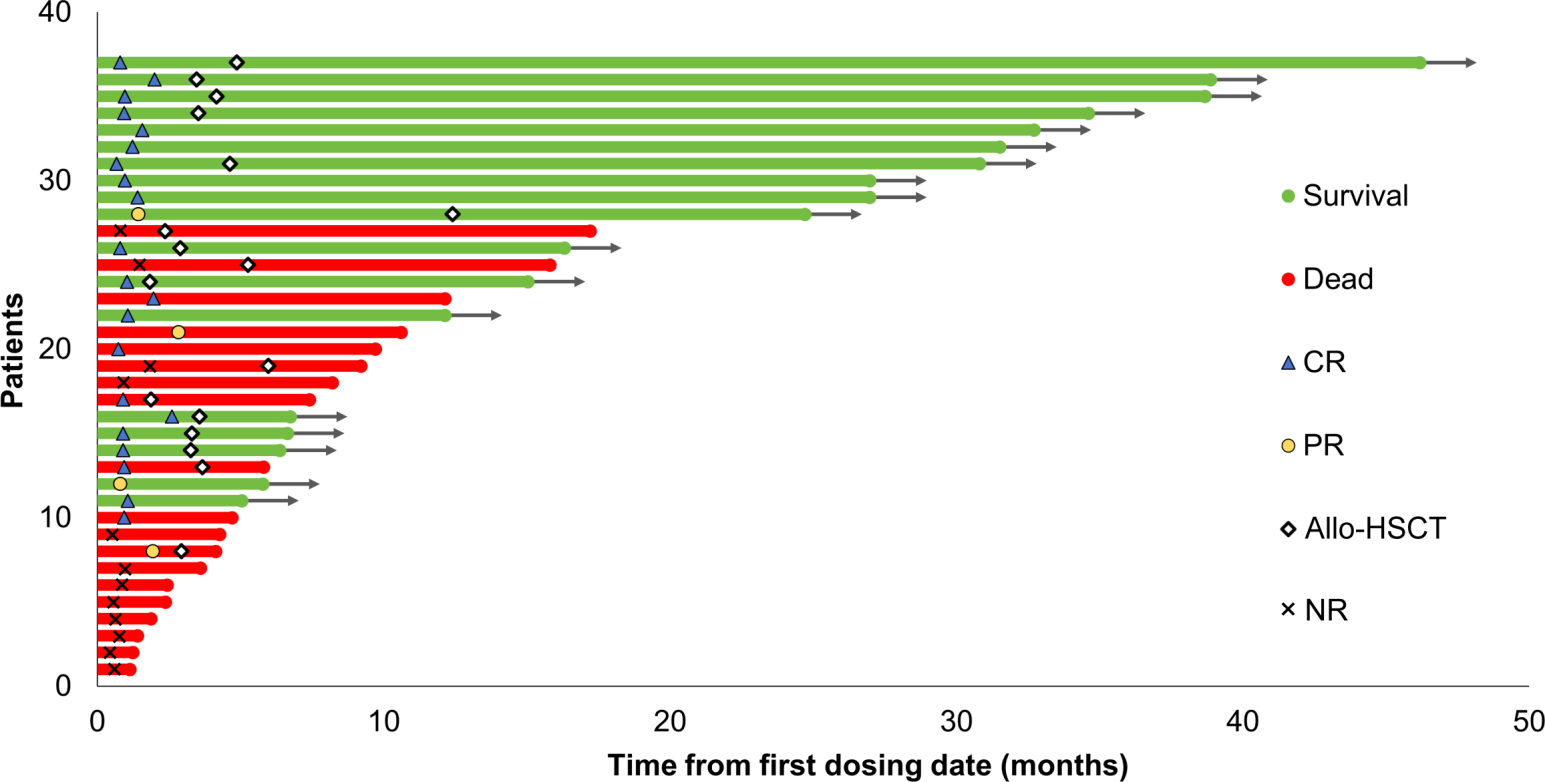
Swimmer plot illustrating the clinical course of patients with relapsed/refractory AML treated with the tislelizumab + HMA + CAG regimen. The best response, survival status, and allo-HSCT status of the enrolled patients are presented. Allo-HSCT, allogeneic hematopoietic stem cell transplantation; CR, complete remission; PR, partial remission; NR, no response; HMA, hypomethylating agent; CAG, decitabine/azacitidine, cytarabine, idarubicin/aclarubicin, G-CSF.

The swimmer’s plot presented in **Figure 1** illustrates the optimal response, allo-HSCT, and survival status of the enrolled patients. All patients completed at least 1 treatment cycle, with 18 completing 2 cycles and 4 completing 3 cycles. The median duration to treatment was 3.3 months (0.9 - 5.5 months). Study discontinuations were not related to protocol but were due to primary refractory disease (n=12), relapse after the initial response (n=3), allo-HSCT in CR/CRi (n=11) and PR (n=1), death (n=1) and patient preference (n=9).

**Figure 2:**
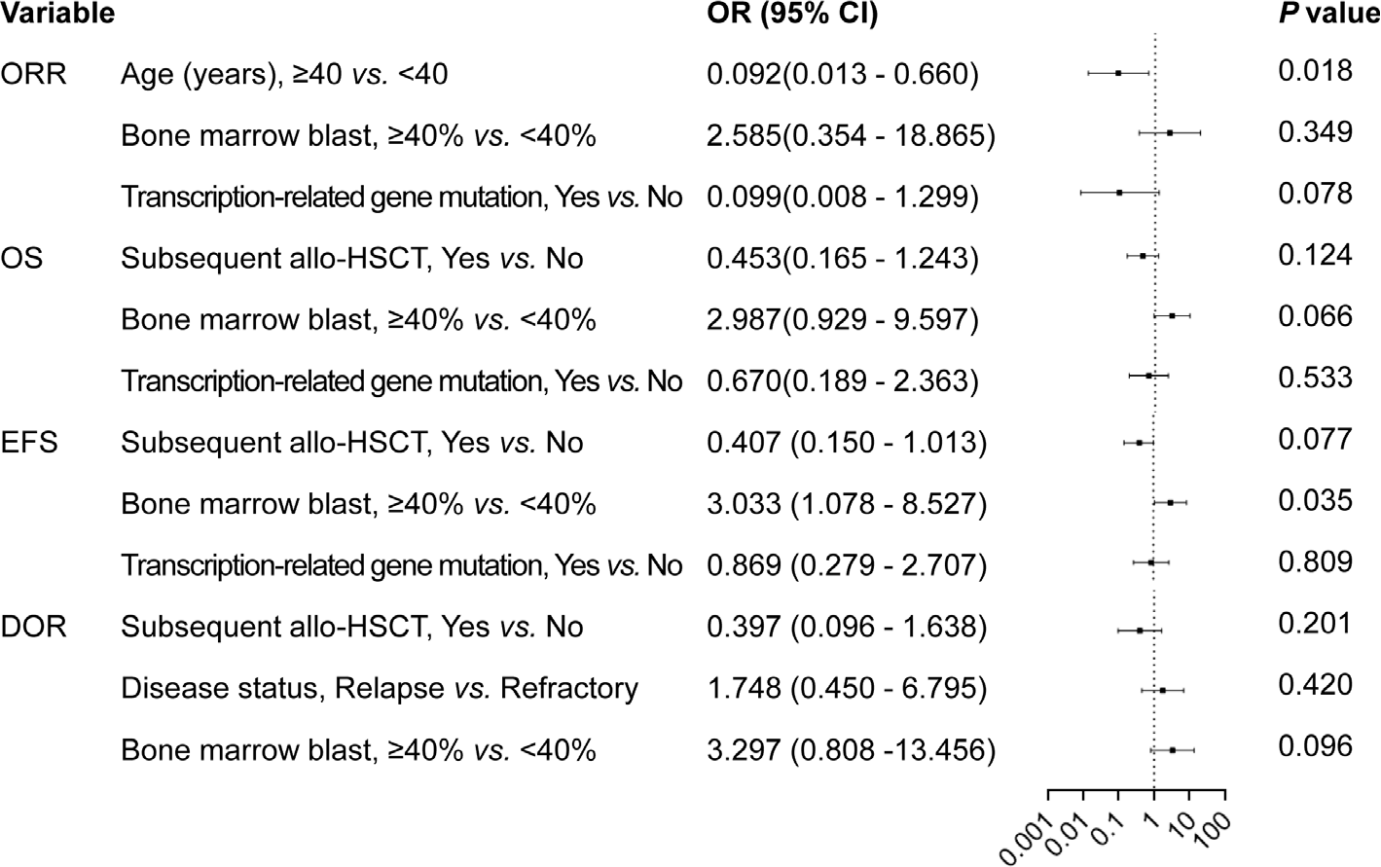
Forest plot illustrating the multivariate analysis of prognostic factors for overall survival, event-free survival, and duration of response in patients with relapsed/refractory AML treated with the tislelizumab + HMA + CAG regimen. Allo-HSCT, allogeneic hematopoietic stem cell transplantation; DOR, duration of response; EFS, event-free survival; OR, Objective response; OS, overall survival; RUNX1, runt-related transcription factor 1; HMA, hypomethylating agent; CAG, decitabine/azacitidine, cytarabine, idarubicin/aclarubicin, G-CSF.

At a median follow-up of 27 months (range, 19.8-34.2 months) for the entire cohort, all 37 patients but 1 were evaluated for response. This included 21 patients who achieved CR/CRi (58.3%), comprising 19 patients with CR and 2 patients with CRi, as well as 4 patients (11.1%) who achieved PR. Additionally, 8 patients (22.2%) achieved MRD-negative CR after the first cycle of treatment, 3 patients achieved MRD-negative CR after the second cycle of treatment, and 2 patients achieved MRD-negative CR after the third cycle of treatment. No response to treatment was observed in 11 patients (30.6%) enrolled in the study. The univariate analysis revealed a significant association between the age group of ≥ 40 years, the presence of transcription-related gene mutations, and a high ORR (*P*=0.020 and *P*=0.025, respectively; **Table 2**). The multivariate analysis identified a significant correlation between the age group of ≥ 40 years and a high (*P*=0.018; **Figure 2**), as well as a marginally significant association between the presence of transcription-related gene mutations and an improved ORR (*P*=0.078; **Figure 2**).

**Table 2:** Univariate analysis of prognostic factors for overall response rate.

Variable	Univariate analysis		*P* value
	Response *n* (%)	No response *n* (%)	
Gender			1.000
Male	17 (68.0)	8 (32.0)	
Female	8 (72.7)	3 (27.3)	
Age (years)			0.020
< 40	5 (41.7)	7 (58.3)	
≥ 40	20 (83.3)	4(16.7)	
Diagnosis			0.631
AML de novo	22 (71.0)	9 (29.0)	
Secondary AML	3 (60.0)	2 (40.0)	
Antecedent allo-HSCT			1.000
No	23 (69.7)	10 (30.3)	
Yes	2 (66.7)	1 (33.3)	
Past HMA exposure			0.214
No	21 (75.0)	7 (25.0)	
Yes	4 (50.0)	4 (50.0)	
Disease status			0.703
Refractory	18 (72.0)	7 (28.0)	
Relapse	7 (63.6)	4 (36.4)	
Bone marrow blast (%)			0.073
< 40	19 (79.2)	5 (20.8)	
≥ 40	6 (50.0)	6 (50.0)	
ELN 2022 risk classification			1.000
Favorable	10 (71.4)	4 (28.6)	
Intermediate/Adverse	15 (68.2)	7 (31.8)	
Normal karyotype			0.888
No	12 (70.6)	5 (29.4)	
Yes	13 (68.4)	6 (42.9)	
DNA methylation-related gene mutations^a^			0.729
No	14 (66.7)	7 (33.3)	
Yes	11 (73.3)	4 (26.7)	
RAS pathway-related gene mutations^a^			0.703
No	18 (72.0)	7 (28.0)	
Yes	7 (63.6)	4 (36.4)	
Transcription-related gene mutations^a^			0.025
No	12 (54.5)	10 (45.5)	
Yes	13 (92.9)	1 (7.1)	
RNA splicing-related gene mutations^a^			1.000
No	21 (70.0)	9 (30.0)	
Yes	4 (66.7)	2 (33.3)	

a DNA methylation-related gene mutation include TET2, DNMT3A, IDH1, IDH2, and WT1. RAS pathway-related gene mutations include KRAS, NRAS, FTL3-ITD, PTPN11, KIT, and CBL. Transcription-related gene mutations include RUNX1, BCOR, CEBPA, and NPM1. RNA splicing-related gene mutations include SRSF2 and U2AF1.

allo-HSCT, allogeneic hematopoietic stem cell transplantation; AML, acute myeloid leukemia; ASXL1, additional sex comb like 1; CEBPA-bZip, CCAAT/enhancer-binding protein α- basic leucine zipper; HMA, hypomethylating agent; CAG, decitabine/azacitidine, cytarabine, idarubicin/aclarubicin, G-CSF; ELN, European LeukemiaNet; FLT3-ITD, FMS-like tyrosine kinase-3 internal tandem duplication; RUNX1, runt-related transcription factor 1;TET2, ten-eleven translocation 2; U2AF1,U2 small nuclear RNA auxiliary factor 1.

Sixty-four consecutive patients with r/r AML who received at least one cycle of treatment with the HMA + CAG regimen at the First Medical Center of Chinese PLA General Hospital in Beijing from January 1, 2012 to December 31, 2022 were included in the historical control group (**Supplementary Figure 1**). To more accurately assess the comparative benefit of incorporating tislelizumab into the HMA + CAG regimen and to minimize the influence of potential confounding factors, propensity score matching (PSM) was employed for group allocation. Following PSM, a total of 68 patients were included in the analysis, comprising 24 patients in the tislelizumab + HMA + CAG group and 44 patients in the HMA + CAG group. No significant differences were observed between groups regarding baseline characteristics (**Supplementary Table 1**). The ORR of the tislelizumab + HMA + CAG group was significantly higher than that of the historical control group (*P*=0.028; **Table 3**). The ORR for the tislelizumab + HMA + CAG group was 87.5%, with 18 patients (75.0%) achieving CR/CRi, and 3 patients (12.5%) achieving PR. In contrast, the ORR for the historical control group was 61.4%, with 11 patients (52.3%) achieving CR/CRi and 4 patients (9.1%) achieving PR. Multivariate analysis identified a significant correlation between AML *de novo* and higher ORR (*P*=0.018; **Supplementary Table 3**), as well as a marginally significant association between the treatment of tislelizumab + HMA + CAG and improved ORR (*P*=0.078; **Supplementary Table 3**). These findings suggest that the results remain robust following PSM.

**Table 3:** Survival and treatment response outcomes for propensity score matched subgroups.

Outcome	Description	Propensity score matched subgroups		*P* value
		Tislelizumab + HMA + CAG (n = 24)	HMA + CAG (n = 44)	
Response	CR/CRi	18 (75.0%)	23 (52.3%)	0.028
	PR	3 (12.5%)	4 (9.1%)	
	NR	3 (12.5%)	17 (38.6%)	
OS	Alive	16 (66.7%)	20 (45.5%)	0.323
	Dead	8 (33.3%)	24 (34.8%)	
	Median months (95% CI)	Not reached	23.3 (4.3 - 42.3)	
EFS	No event	14 (58.3%)	10 (22.7%)	0.026
	Relapse/death	10 (41.7%)	34 (77.3%)	
	Median months (95% CI)	Not reached	5.1 (0 - 13.7)	
DOR	No event	14(66.7%)	11 (40.7%)	0.551
	Relapse/death	7 (33.3%)	14 (59.3%)	
	Median months (95% CI)	Not reached	19 (2.3 - 35.7)	
60-day mortality	Alive	23 (95.8%)	39 (88.6%)	0.413
	Dead	1(4.2%)	5 (11.4%)	

CR, complete remission; CRi, CR with incomplete hematologic recovery; CI, confidence interval; HMA, hypomethylating agent; CAG, decitabine/azacitidine, cytarabine, idarubicin/aclarubicin, G-CSF; DOS, duration of response; EFS, event-free survival; PR, partial remission; NR, no response; OS, overall survival.

### 3.3 Survival and duration of response

Among the 37 patients in the tislelizumab + HMA + CAG group, the overall mortality rate was 54.1% (n=20), with a 60-day mortality rate of 10.8% (n=4). One patient died from a lung infection without BM assessment on day 34 post-treatment, while three patients succumbed to rapid disease progression combined with lung infection on days 27, 37, and 56 post-treatment, respectively. Sixteen patients (43.2%) died after discontinuing therapy: twelve due to r/r AML, three due to post-transplant complications, and one from cardiac arrest.

**Figure 3:**
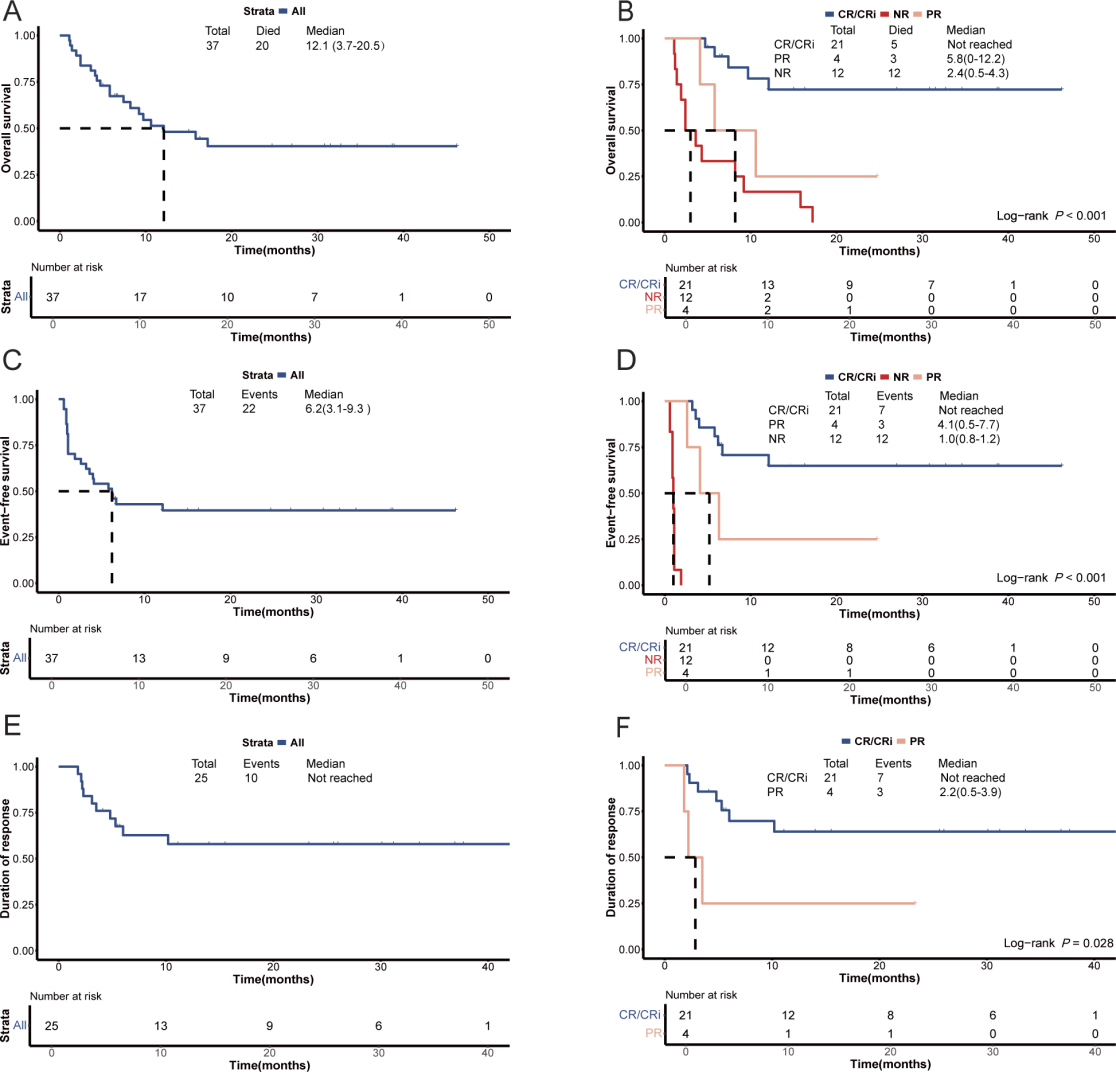
Survival plots for patients with relapsed/refractory AML treated with the tislelizumab + HMA + CAG regimen. **(A)** Overall survival of the enrolled patients. **(B)** Comparison of overall survival between patients who achieved an objective response and those who did not respond. Comparison of overall survival between patients who achieved CR/CRi and those who achieved PR. **(C)** Event-free survival of the enrolled patients. **(D)** Comparison of event-free survival between patients who achieved an objective response and those who did not respond. Comparison of event-free survival between patients who achieved CR/CRi and those who achieved PR. **(E)** Duration of response among patients who attained an objective response. **(F)** Comparison of duration of response between patients with an objective response. CR, complete remission; CRi, CR with incomplete hematologic recovery; PR, partial remission; NR, no response; HMA, hypomethylating agent; CAG, decitabine/azacitidine, cytarabine, idarubicin/aclarubicin, G-CSF.

The median OS of the patients was 12.1 months (95% CI, 3.7-20.5 months), with a median EFS and DOR of 6.2 months (95% CI 3.1-9.3 months) and not reached, respectively (**Figures 3A, C, E**). Responders (CR/CRi + PR) exhibited a significantly longer median OS compared with non-responders (not reached *vs*. 2.4 months [95% CI, 0.5-4.3 months], *P*<0.001; **Figure 3B**). Among responders, those who achieved CR/CRi had a significantly longer median OS compared with those who achieved PR (not reached *vs*. 5.8 months [95% CI, 0-12.2 months], *P*=0.032; **Figure 3B**). Moreover, responders had a longer median EFS compared with non-responders (not reached *vs*. 1.0 months [95% CI, 0.8-1.2 months], *P*<0.001; **Figure 3D**). The median EFS of patients who achieved CR/CRi was slightly higher than those who achieved PR among the responders (not reached *vs*. 4.1 months [95% CI, 0.5-7.7 months], *P*=0.067; **Figure 3D**), with a marginal statistical significance. Furthermore, the median DOR was significantly longer for those achieving CR/CRi compared with those achieving PR among the responders (not reached *vs*. 2.2 months [95% CI, 0.5-3.9 months], *P*=0.028; **Figure 3F**). Univariate analysis indicated that subsequent allo-HSCT, and BM blast percentage less than 40% were significantly associated with improved OS (*P*=0.018 and *P*=0.001, respectively; **Supplementary Figure 2**), EFS (*P*=0.006 and *P*<0.001, respectively; **Supplementary Figure 3**), and DOR (*P*=0.043 and *P*=0.003, respectively; **Supplementary Figure 4**) (**Supplementary Table 2**). In addition, univariate analysis revealed that carrying mutations in transcription-related genes was significantly associated with a superior OS (*P*=0.035; **Supplementary Figure 2**), weakly correlated with a better EFS (*P*=0.064; **Supplementary Figure 3**), but showed no association with the DOR (*P*>0.050; **Supplementary Figure 4**) (**Supplementary Table 2**). Multivariate analysis revealed a significant correlation between BM blast <40% and improved EFS (*P*=0.035; **Figure 2**). Additionally, multivariate analysis showed a marginally significant association between BM blast percentage less than 40% and better OS (*P*=0.066; **Figure 2**) and DOR (*P*=0.096; **Figure 2**). Furthermore, multivariate analysis demonstrated a marginally significant association between receiving subsequent allo-HSCT and better EFS (*P*=0.077; **Figure 2**).

**Figure 4:**
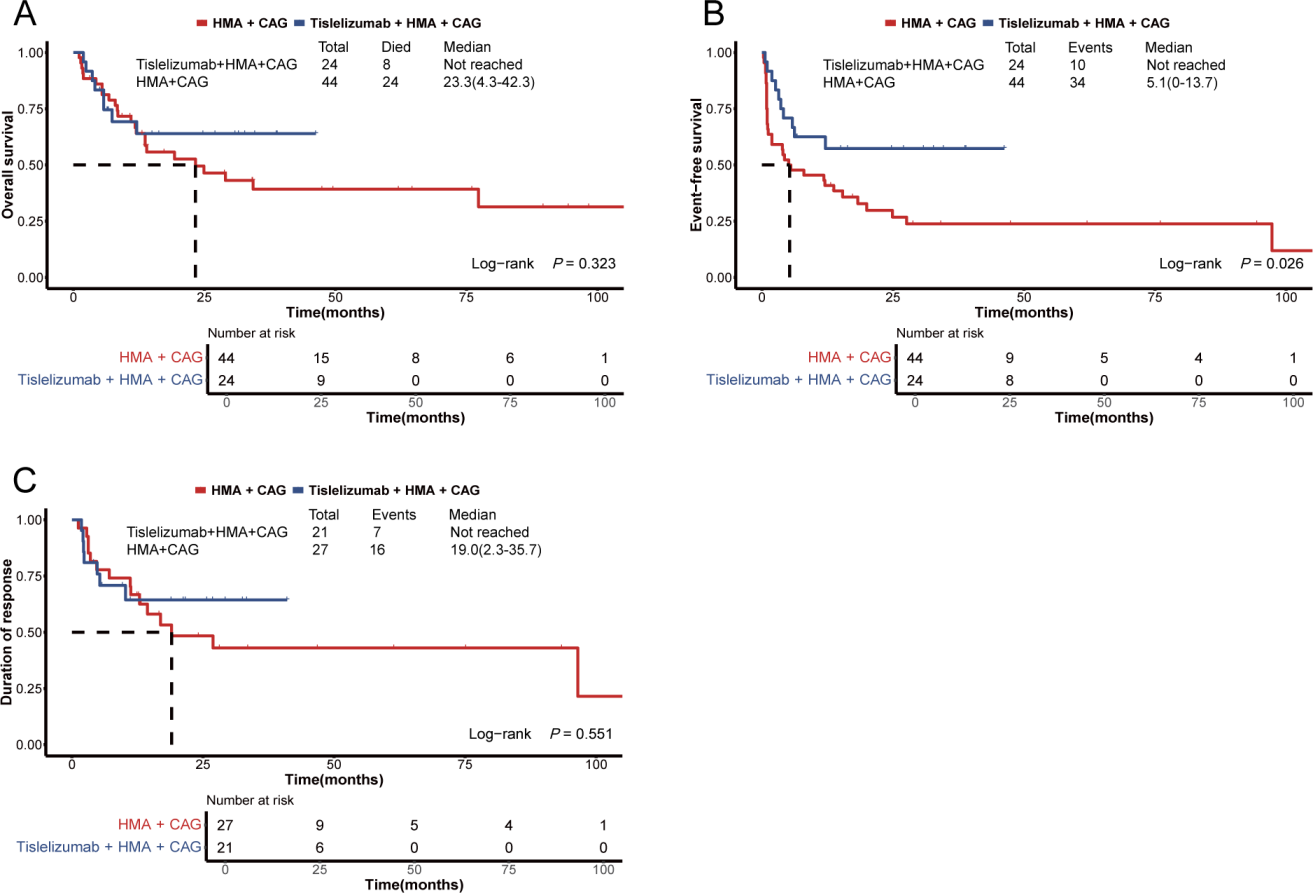
Survival plots for patients with relapsed/refractory AML treated with the HMA + CAG regimen, combined with or without tislelizumab. **(A)** Overall survival of patients enrolled in the propensity score-matched subgroup. **(B)** Event-free survival of patients enrolled in the propensity score-matched subgroup. **(C)** Duration of response among patients who achieved an objective response within the propensity score-matched subgroup. HMA, hypomethylating agent; CAG, decitabine/azacitidine, cytarabine, idarubicin/aclarubicin, G-CSF.

Following PSM, the 60-day mortality rate among patients in the tislelizumab + HMA + CAG group did not demonstrate a statistically significant difference compared to the historical control group (4.2% *vs*. 11.4%, *P*=0.413; **Table 3**). The median OS and DOR for patients in the tislelizumab + HMA + CAG group were not significantly longer than those observed in the historical control group (not reached *vs*. 23.3 months [95% CI, 4.3-42.3 months], *P*=0.323; not reached *vs*. 19.0 months [95% CI, 2.3-35.7 months], *P*=0.551; **Table 3** and **Figure 4**). However, the median EFS for the tislelizumab + HMA + CAG group was significantly superior to that of the historical control group (not reached *vs*. 5.1 months [95% CI, 0-13.7 months], *P*=0.026; **Table 3** and **Figure 4**).

Seventeen patients (45.9%) underwent allo-HSCT after the tislelizumab + HMA + CAG regimen treatment. The donors for these transplants included human leukocyte antigen haploidentical sibling donors (n=12) and human leukocyte antigen-matched sibling donors (n=5). The median interval from the last administration of tislelizumab + HMA + CAG therapy to transplantation was 71 days (range, 44-208 days). Among patients undergoing allo-HSCT, 6/17 (35.3%) developed acute graft-versus-host disease (aGVHD), with a median onset at +19 days post-transplant (range, 12-24 days). These included cases of grade 1 GVHD (skin GVHD, n=2), grade 2 GVHD (skin GVHD, n=1; upper gastrointestinal tract GVHD, n=1), and grades 3-4 GVHD (lower gastrointestinal tract GVHD, n=2). The 1-year incidence of chronic GVHD (cGVHD) was 14.8% (95%CI 2.1%-38.8%) and all patients presented with mild cGVHD. By the time of follow-up, 6 patients had died after transplant, whereas 11 patients were still alive and in remission between 2.8 to 41.3 months post-transplant. With a median follow-up time of 26.1 months (range, 5.5 - 46.7 months), the 1-year and 2-year OS rates of patients who underwent allo-HSCT were 66.4% (95% CI, 37.0%-84.7%) and 55.5% (95% CI, 24.7%-78.1%), respectively. Of patients who passed away after the transplant, 2 died due to infections after day 63 and day 445; 1 died due to grade 4 gastrointestinal tract GVHD after day 36; 2 died due to relapse after day 166 and day 317; and 1 died due to progressive AML after day 98.

### 3.4 Adverse events

No treatment discontinuation occurred because of AEs with tislelizumab + HMA + CAG treatment. Hematologic toxicities were observed in all patients who received treatment with the tislelizumab + HMA + CAG regimen. This included a high incidence of grade 4 thrombocytopenia (80.7%) and neutropenia (82.5%), followed by grade 3 anemia (77.2%). AEs associated with infection were also observed, with high frequencies of lung infection (26.3%) and grade 3 febrile neutropenia (66.7%). In addition, 5 patients (8.5%) experienced grades 2-3 immune-related adverse events (irAEs) and no deaths were directly attributable to irAEs (**Table 4**). After PSM, there were no significant differences between the tislelizumab + HMA + CAG group and the historical control group in the incidences of grade 3-4 infection-related events (83.7% *vs*. 83.3%, *P*=0.958) and the incidence of lung infection (18.6% *vs*. 26.7%, *P*=0.340). The detailed incidence of AEs is provided in **Supplementary Tables 4**, **5**.

**Table 4:** Hematologic and nonhematologic treatment-related toxicities of all cycles^*^.

Adverse event	Grade					Total
	5	4	3	2	1	
Immune-related adverse events						
Immune-related thyroiditis			1 (1.7)			1 (1.7)
Immune-related pneumonitis			1 (1.7)			1 (1.7)
Temporomandibular arthritis				1 (1.7)		1 (1.7)
Rash maculo-papular				2 (3.4)		2 (3.4)
Infection						
Febrile neutropenia			38 (64.4)			38 (64.4)
Lung infection	1 (1.7)		14 (23.7)			15 (25.4)
Sepsis			7 (11.9)			7 (11.9)
Small intestine infection			2 (3.4)			2 (3.4)
Urinary tract infection			1 (1.7)			1 (1.7)
Anal mucositis			1 (1.7)			1 (1.7)
Hepatitis B reactivation				1 (1.7)		1 (1.7)
Hematologic treatment-related toxicities						
Neutropenia		47 (79.7)	3 (5.1)		1 (1.7)	51 (86.4)
Thrombocytopenia		46 (78.0)	4 (6.8)		1 (1.7)	51 (86.4)
Anemia			44 (74.6)	7 (11.9)		51 (86.4)
Nonhematologic treatment-related toxicities						
Alanine aminotransferase increased			1 (1.7)	1 (1.7)	1 (1.7)	3 (5.1)
Alkaline phosphatase increased				1 (1.7)	1 (1.7)	2 (3.4)
Aspartate aminotransferase increased			1 (1.7)	1 (1.7)		2 (3.4)
Blood bilirubin increased			1 (1.7)		5 (8.5)	6 (10.2)
Blood lactate dehydrogenase increased					7 (11.9)	7 (11.9)
Gamma-glutamyltransferase increased			4 (6.8)	4 (6.8)	5 (8.5)	13 (22.0)
Hyperglycemia					8 (13.6)	8 (13.6)
Hyperkalemia					1 (1.7)	1 (1.7)
Hyperphosphatemia					6 (10.2)	6 (10.2)
Hyperuricemia					2 (3.4)	2 (3.4)
Hypoalbuminemia				7 (11.9)	6 (10.2)	13 (22.0)
Hypocalcemia					4 (6.8)	4 (6.8)
Hypoglycemia					2 (3.4)	2 (3.4)
Hypokalemia		1 (1.7)	1 (1.7)	4 (6.8)	12 (20.3)	18 (30.5)
Hypomagnesemia					2 (3.4)	2 (3.4)
Hyponatremia					1 (1.7)	1 (1.7)
Hypophosphatemia				1 (1.7)	7 (11.9)	8 (13.6)
Lower gastrointestinal hemorrhage					1 (1.7)	1 (1.7)
Pericardial effusion				6 (10.2)		6 (10.2)
Pleural effusion					6 (10.2)	6 (10.2)

All data are shown as number of patients (%). ^*^The addition of patients attending each treatment visits: 37 + 18 + 4 = 59.

## 4 Discussion

In this study, our objective was to assess the efficacy and safety of tislelizumab in combination with the HMA plus CAG regimen in a larger cohort. Following an expansion of the initial trial, the total number of patients increased from 27 to 37, resulting in an improvement in ORR from 63% to 69.4%. Moreover, the median follow-up time was extended from 8.2 to 27 months, allowing for observation of long-term effects and leading to an increase in median OS from 9.7 to 12.1 months. The combination of tislelizumab and HMA + CAG regimen demonstrated safety with reported irAEs being mild and low-grade. Following PSM adjustment, the combination therapy strategy demonstrated a significant improvement in ORR and EFS compared to historical controls, indicating its potential for enhancing outcomes in patients with r/r AML. The combination of pembrolizumab and cytarabine for the treatment of r/r AML is currently undergoing a phase II clinical trial. Preliminary results demonstrate that this combination therapy is both effective and safe, with an ORR of 46% and a median OS of 11.1 months (18). These findings are consistent with our preliminary data. The above evidence emphasizes the promising role of PD-1 antibody-based combination therapy in the management of r/r AML and provide valuable insights for future research and clinical practice in this complex disease.

In this study, we observed that 11 patients exhibited a persistent lack of response to the tislelizumab and HMA + CAG regimen. This treatment resistance may be mechanistically linked to tolerance or acquired resistance to immune checkpoint inhibitors (ICI). The multifactorial nature of such resistance encompasses impaired antigen presentation machinery, dysregulation of IFN-γ signaling cascades, neoantigen depletion, and immunosuppressive tumor microenvironment modulation (19, 20). Comprehensive analysis of clinical characteristics associated with therapeutic response may facilitate the identification of patient subpopulations likely to derive clinical benefit from this therapeutic regimen. We discovered that the response rate of patients aged 40 or above was higher than that of patients below 40, which is in line with our previous study (15). Furthermore, age was an independent factor influencing the response rate in the multivariate analysis. Our findings align closely with previously reported observations indicating that older patients with metastatic melanoma respond more favorably to immunotherapy compared to their younger counterparts (21, 22). The underlying reasons for this disparity remain elusive; however, it has been hypothesized that beyond a certain age, the deceleration of tumor growth may indirectly facilitate the ability of immune cells to eliminate tumors (23). Additionally, it is plausible that younger patients may harbor a greater presence of immunosuppressive cells within their tumors, although this remains unverified (22). However, despite the high response rate in patients aged 40 or above in our study, it does not necessarily mean that these patients have a favorable prognosis and survival, indicating that the administration of consolidation therapy post-remission holds greater significance in determining patient survival. Our findings support the notion that undergoing allo-HSCT following remission can extend EFS for patients, emphasizing the importance of timely consideration for eligible individuals.

Our findings further substantiate that those patients with a lower leukemia burden exhibited significantly higher response rates. This enhancement was particularly evident in patients with less than 40% bone marrow blast cells prior to treatment initiation, resulting in improved OS and EFS compared to those with a higher tumor load. These results underscore the importance of personalized approaches tailored to individual patient profiles based on their specific disease presentations, highlighting the potential benefits of targeting patients with lower disease burdens for improved clinical outcomes.

Our analysis on the impact of genetic risk stratification by ELN 2022 at initial diagnosis of AML on treatment response rates and survival outcomes revealed that there were no significant differences in treatment response rates and survival outcomes between patients from the favorable risk group and patients from the high-risk or intermediate-risk group after treatment with tislelizumab + HMA + CAG regimen. This suggests that the prognosis of patients receiving this therapy may not be influenced by genetic risk stratification at initial diagnosis of AML. Notably, our findings indicate that patients harboring mutations in transcription-related genes (RUNX1, CEBPA, BCOR and NPM1) who underwent combination therapy incorporating PD-1 antibodies exhibited a significantly higher response rate. RUNX1 and CEBPA are key transcription factors in the hematopoietic process, and they have the ability to bring TET2 demethylase to its binding site, leading to DNA demethylation nearby. Mutations in RUNX1 and CEBPA in AML patients can affect the methylation status of key regulatory sites, causing suppression of multiple target genes regulated by these transcription factors, which is likely to be achieved through a TET2-dependent mechanism (24). Other studies have found that CEBPA mutations can promote high DNA methylation by relieving the inhibition of DNA methyltransferase 3A, which is an essential enzyme in adding methyl groups to DNA (25). Treatment of leukemia cells with decitabine induced a dose-dependent upregulation of PD-L1, PD-L2, and PD-1 expression (12). Therefore, it can be hypothesized that the aberrant hypermethylation induced by CEBPA and RUNX1 mutations may demonstrate increased sensitivity to demethylation therapy. Additionally, DNA demethylation could potentially enhance the effectiveness of PD-1 blockade in anti-tumor immunotherapy by upregulating the expression of PD-1/PD-L1. Further investigation is warranted to ascertain whether the inhibition of CEBPA and RUNX1 mutations can activate tumor immunity.

In this study, the ORR, OS, and EFS of the 8 patients who had previously received HMAs exhibited a trend toward being lower compared to those who had not received HMAs. However, none of these differences reached statistical significance. These findings suggest that even for patients with AML who have prior exposure to HMAs, this treatment regimen may still provide potential clinical benefits.

The tislelizumab + HMA + CAG regimen was generally deemed to be well-tolerated. Two patients experienced grade 3 irAEs (pneumonitis and thyroiditis). Out of the 5 patients with irAEs, one patient achieved complete remission of leukemia following treatment, while the remaining four patients did not respond to chemotherapy. This aligns with previous studies on solid tumors which found no indication of a causal relationship between irAEs and improved response to PD-1 inhibitor therapy (26). In this study, all patients experienced grades 3-4 myelosuppression after therapy, but showed improvement with active symptomatic support therapy. The elevated incidence of GVHD following allo-HSCT in patients receiving anti-PD-1 therapy constitutes a substantial clinical challenge (27). Current consensus guidelines recommend a 6-week washout period between PD-1 blockade therapy and allo-HSCT, based on the prolonged serum and tissue half-life of anti-PD-1 antibodies to mitigate risks of GVHD (27). A clinical investigation involving Hodgkin lymphoma patients receiving allo-HSCT post-anti-PD-1 therapy demonstrated a direct correlation between extended treatment-transplantation intervals and reduced GVHD incidence (28). Further, our research group has previously established the efficacy of post-transplantation cyclophosphamide (PTCy) in GVHD prevention for ICI-treated patients (29, 30). The protective mechanism of PTCy-based prophylaxis appears to involve T-cell subset modulation, potentially restoring immune homeostasis (31, 32). In our cohort, all 17 patients maintained >6-week intervals between final anti-PD-1 administration and allo-HSCT, with 6 subjects receiving PTCy prophylaxis - factors potentially contributing to reduced GVHD risk. Notably, the tislelizumab + HMA + CAG regimen cohort exhibited a cumulative grade 3-4 acute GVHD incidence of 11.8%, with a single fatal GVHD event and no moderate-to-severe chronic GVHD cases. Future investigations should prioritize elucidation of ICI immunomodulatory mechanisms and identification of predictive biomarkers for ICI-associated GVHD, which would facilitate improved risk management of pre-transplant immunotherapy and refinement of therapeutic algorithms for GVHD mitigation.

This single-arm study was conducted at a single center, and due to the inability to conduct randomized control, only a historical control study could be carried out. In historical control studies, time bias is a common concern that may arise from factors such as evolving diagnostic criteria, changing treatment protocols, variations in patient population characteristics, and shifting standards of care over time. To mitigate this potential time bias, we employed PSM. After PSM adjustment for variables that might affect survival, censoring of some patients resulted in a reduction in the sample size. Insufficient patient numbers in certain subgroups may lead to biased or overinterpreted conclusions from subgroup analyses. The relatively short follow-up period for the tislelizumab + HMA + CAG group compared with the historical control group may have impacted the accuracy of survival and prognosis analysis between the two groups. Therefore, further confirmation through multicenter exploration and long-term follow-up data is necessary to validate the results of this trial.

In summary, this study provided more comprehensive data for evaluating tislelizumab combined with the tislelizumab + HMA + CAG regimen by increasing the sample size and extending the follow-up time. These data not only help understand the potential of this treatment combination in r/r AML but also lay the foundation for future research in this field. Furthermore, with the accumulation of more data, it is expected to reveal the differences between different treatment strategies, thereby promoting personalized medicine and improving outcomes of patients with r/r AML.

## Data Availability

The original contributions presented in the study are included in the article/**Supplementary Material**. Further inquiries can be directed to the corresponding authors.
