# Supplementary material for: Updates from a single-center phase 2 study of PD-1 inhibitor combined with hypomethylating agent plus CAG regimen in patients with relapsed/refractory acute myeloid leukemia
Source: Front Immunol. 2025 Apr 17;16:1533467. doi: 10.3389/fimmu.2025.1533467 (PMC12043577; doi:10.3389/fimmu.2025.1533467)
Supplement: Supplementary file 1 [file DataSheet1.docx]

Supplementary Material

**Updates from a single-center phase 2 study of PD-1 inhibitor combined with hypomethylating agent plus CAG regimen in patients with relapsed/refractory acute myeloid leukemia**

**Supplementary Figures and Tables**

**1.1 Supplementary Tables**

**Supplementary Table 1** Baseline characteristics of patients with AML in propensity score matched subgroups

| Variable | Propensity score matched subgroup | | | |
| --- | --- | --- | --- | --- |
|  | Total | Tislelizumab + HMA + CAG | HMA + CAG | *P* value |
| Total number of patients | 68 | 24 | 44 |  |
| Age, median, years (range) | 44 (12 - 79) | 44 (18 - 71) | 44 (12 - 79) | 0.664 |
| Gender |  |  |  | 0.428 |
| Male | 41 (63.0%) | 16 (66.7%) | 25 (56.8%) |  |
| Female | 27 (37.0%) | 8 (33.3%) | 19(43.2%) |  |
| Antecedent allo-HSCT |  |  |  | 0.413 |
| No | 62 (89.1%) | 23 (95.8%) | 39 (88.6%) |  |
| Yes | 6 (10.9%) | 1 (4.2%) | 5(11.4%) |  |
| Past HMA exposure |  |  |  | 0.710 |
| No | 50 (73.5%) | 17 (70.8%) | 33 (75.0%) |  |
| Yes | 18 (26.5%) | 7 (29.2%) | 11 (25.0%) |  |
| Subsequent allo-HSCT |  |  |  | 0.695 |
| No | 29 (42.6%) | 11 (45.8%) | 18 (40.9%) |  |
| Yes | 39 (57.4%) | 13 (54.2%) | 26 (59.1%) |  |
| Diagnosis |  |  |  | 1.000 |
| AML *de novo* | 59 (86.8%) | 21 (87.5%) | 38 (86.4%) |  |
| Secondary AML | 9 (13.2%) | 3 (12.5%) | 6 (13.6%) |  |
| Disease status |  |  |  | 0.678 |
| Refractory | 46 (67.6%) | 17 (70.8%) | 29 (65.9%) |  |
| Relapse | 22 (32.4%) | 7 (29.2%) | 15 (34.1%) |  |
| Bone marrow blast, median, % (range) | 19 (5.0 - 94.4) | 24.5 (5.6 - 83.6) | 16.6 (5.0 - 94.4) | 0.801 |
| White blood cell count, median, × 10^9^/L (range) | 5.9 (0.6 -172.1) | 3.5 (0.6 - 80.8) | 8.8 (0.6 - 172.1) | 0.183 |
| Platelets, median, × 10^9^/L (range) | 99 (14 - 521) | 116 (16 - 310) | 83 (14 - 521) | 0.207 |
| Hemoglobin, median, g/L (range) | 83 (30 - 140) | 76 (37 - 140) | 87 (30 - 130) | 0.197 |
| ELN 2022 risk classification |  |  |  | 0.079 |
| Favorable | 17 (25.0%) | 9 (37.5%) | 8 (18.2%) |  |
| Intermediate/Adverse | 51 (75.0%) | 15 (65.2%) | 36 (81.8%) |  |
| Normal karyotype |  |  |  | 0.649 |
| No | 28 (41.2%) | 9 (37.5%) | 19 (43.2%) |  |
| Yes | 40 (58.8%) | 15 (62.5%) | 25 (56.8%) |  |
| DNA methylation-related gene mutations^a^ |  |  |  | 0.252 |
| No | 43 (63.2%) | 13 (54.2%) | 30 (68.2%) |  |
| Yes | 25 (36.8%) | 11 (45.8%) | 14 (31.8%) |  |
| RAS pathway-related gene mutations^a^ |  |  |  | 0.335 |
| No | 49 (71.2%) | 19 (79.2%) | 30 (68.2%) |  |
| Yes | 19 (27.9%) | 5 (20.8%) | 14 (31.8%) |  |
| Transcription-related gene mutations^a^ |  |  |  | 0.140 |
| No | 42 (61.8%) | 12 (50.0%) | 30 (68.2%) |  |
| Yes | 26 (38.2%) | 12 (50.0%) | 14 (31.8%) |  |
| RNA splicing-related gene mutations^a^ |  |  |  | 0.439 |
| No | 60 (88.2%) | 20 (83.3%) | 40 (90.9%) |  |
| Yes | 8 (11.8%) | 4 (16.7%) | 4 (9.1%) |  |

^a^DNA methylation-related gene mutation include TET2, DNMT3A, IDH1, IDH2, and WT1. RAS pathway-related gene mutations include KRAS, NRAS, FTL3-ITD, PTPN11, KIT, and CBL. Transcription-related gene mutations include RUNX1, BCOR, CEBPA, and NPM1. RNA splicing-related gene mutations include SRSF2 and U2AF1.

Allo-HSCT, allogeneic hematopoietic stem cell transplantation AML; AML, acute myeloid leukemia; HMA, hypomethylating agent; CAG, decitabine/azacitidine, cytarabine, idarubicin/aclarubicin, G-CSF; ELN, European LeukemiaNet; FLT3-ITD, FMS-like tyrosine kinase-3 internal tandem duplication; U2AF1, U2 small nuclear RNA auxiliary factor 1 gene.

**Supplementary Table 2** Overall survival, event-free survival and duration of response by baseline characteristics in refractory and relapse patients

| Variable | 12 - month OS | |  | 12 - month EFS | |  | 12 - month DOR | |
| --- | --- | --- | --- | --- | --- | --- | --- | --- |
|  | % (95% CI) | *P* value |  | % (95% CI) | *P* value |  | % (95% CI) | *P* value |
| Gender |  | 0.554 |  |  | 0.821 |  |  | 0.477 |
| Male | 54.81 (33.07 - 72.12) |  |  | 45.45 (16.66 - 70.69) |  |  | 45.00 (10.76 - 75.13) |  |
| Female | 43.64 (14.70 - 69.86) |  |  | 41.54 (22.55 - 59.56) |  |  | 63.53 (37.57 - 81.81) |  |
| Age (years) |  | 0.530 |  |  | 0.440 |  |  | 0.102 |
| <40 | 53.33 (19.88 - 78.30) |  |  | 41.46 (15.25 - 66.53) |  |  | 73.56 (52.21 - 86.50) |  |
| ≥40 | 50.67 (29.71 - 68.33) |  |  | 44.00 (24.49 - 61.94) |  |  | 51.52 (33.54 - 66.85) |  |
| Diagnosis |  | 0.827 |  |  | 0.591 |  |  | 0.823 |
| AML *de novo* | 51.05 (31.37 - 67.71) |  |  | 44.67 (26.78 - 61.07） |  |  | 56.34 (32.43 - 74.66） |  |
| Secondary AML | 50.00 (11.09 - 80.37) |  |  | 33.33 (4.61 - 67.56） |  |  | 66.67 (5.41 - 94.52） |  |
| Antecedent allo-HSCT |  | 0.583 |  |  | 0.409 |  |  | 0.274 |
| No | 49.66 (31.17 - 65.69) |  |  | 40.72 (24.24 - 56.58) |  |  | 53.89 (30.91 - 72.22) |  |
| Yes | 66.67 (5.41 - 94.52) |  |  | 66.67 (5.41 - 94.52) |  |  | 100 |  |
| Past HMA exposure |  | 0.421 |  |  | 0.191 |  |  | 0.844 |
| No | 43.44 (23.18 - 62.16) |  |  | 45.83 (26.83 - 62.97) |  |  | 60.03 (35.4 - 77.82) |  |
| Yes | 33.33 (7.83 - 62.26) |  |  | 22.22 (3.37 - 51.31) |  |  | 50.00 (5.78 - 84.49) |  |
| Subsequent allo-HSCT |  | 0.018 |  |  | 0.006 |  |  | 0.043 |
| No | 33.00 (13.81 - 53.75) |  |  | 25.00 (9.10 - 44.85) |  |  | 36.36 (11.18 - 62.68) |  |
| Yes | 73.53 (43.43 - 89.28) |  |  | 64.71 (37.71 - 82.34) |  |  | 77.38 (44.93 - 92.11) |  |
| Disease status |  | 0.185 |  |  | 0.122 |  |  | 0.069 |
| Refractory | 58.46 (36.09 - 75.37） |  |  | 49.36 (29.09 - 66.78) |  |  | 70.71 (42.87 - 86.77) |  |
| Relapse | 36.36 (11.18 - 62.68） |  |  | 27.27 (6.52 - 53.89) |  |  | 28.57 (4.11 - 61.15) |  |
| Bone marrow blast (%) |  | 0.001 |  |  | <0.001 |  |  | 0.003 |
| < 40 | 69.27 (45.86 - 84.12) |  |  | 61.90 (39.42 - 78.11) |  |  | 70.98 (43.32 - 86.89) |  |
| ≥ 40 | 18.46 (3.06 - 44.09) |  |  | 7.69 (0.48 - 29.20) |  |  | 16.67 (0.77 - 51.68) |  |
| ELN 2022 risk classification |  | 0.778 |  |  | 0.865 |  |  | 0.495 |
| Favorable | 72.73 (37.08 - 90.28) |  |  | 35.71 (13.03 - 59.44) |  |  | 50.00 (18.36 - 75.32) |  |
| Intermediate/Adverse | 51.97 (28.50 - 71.09) |  |  | 47.83 (26.83 - 66.13) |  |  | 63.64 (32.69 - 83.30) |  |
| Normal karyotype |  | 0.535 |  |  | 0.958 |  |  | 0.917 |
| No | 38.89 (17.49 - 59.96) |  |  | 38.89 (17.49 - 59.96) |  |  | 58.33 (27.01 - 80.09) |  |
| Yes | 67.67 (41.63 - 84.03) |  |  | 47.37 (24.44 - 67.28) |  |  | 56.98 (24.25 - 79.99) |  |
| DNA methylation-related gene mutations^a^ |  | 0.821 |  |  | 0.890 |  |  | 0.652 |
| No | 51.95 (28.91 - 70.78) |  |  | 40.91 (20.85 - 60.07) |  |  | 64.29 (34.33 - 83.31) |  |
| Yes | 51.43 (23.78 - 73.50) |  |  | 45.71 (20.10 - 68.26) |  |  | 47.73 (15.54 - 74.54) |  |
| RAS pathway-related gene mutations^a^ |  | 0.573 |  |  | 0.555 |  |  | 0.169 |
| No | 47.64 (27.20 - 65.55) |  |  | 38.08 (19.97 -56.06) |  |  | 48.89 (24.60 - 69.41) |  |
| Yes | 60.61 (25.07 - 83.42) |  |  | 54.55 (22.85 - 77.96) |  |  | 85.71 (33.41 - 97.86) |  |
| Transcription-related gene mutations^a^ |  | 0.035 |  |  | 0.064 |  |  | 0.950 |
| No | 37.74 (18.25 - 58.93) |  |  | 34.78 (16.63 - 53.71) |  |  | 55.56 (23.11 - 78.99) |  |
| Yes | 70.71 (39.38 - 87.91) |  |  | 57.14 (28.40 - 77.97) |  |  | 59.83 (28.50 - 81.04) |  |
| RNA splicing-related gene mutations^a^ |  | 0.216 |  |  | 0.677 |  |  | 0.525 |
| No | 44.14 (25.36 - 61.42) |  |  | 41.40 (24.03 - 57.96) |  |  | 54.03 (29.74 - 73.15) |  |
| Yes | 83.33 (27.31 - 97.47) |  |  | 50.00 (11.09 - 80.37) |  |  | 75.00 (12.79 - 96.05) |  |

^a^DNA methylation-related gene mutation include TET2, DNMT3A, IDH1, IDH2, and WT1. RAS pathway-related gene mutations include KRAS, NRAS, FTL3-ITD, PTPN11, KIT, and CBL. Transcription-related gene mutations include RUNX1, BCOR, CEBPA, and NPM1. RNA splicing-related gene mutations include SRSF2 and U2AF1.

Allo-HSCT, allogeneic hematopoietic stem cell transplantation; AML, acute myeloid leukemia; CI, confidence interval; ASXL, additional sex combs like; HMA, hypomethylating agent; CAG, decitabine/azacitidine, cytarabine, idarubicin/aclarubicin, G-CSF; DOR, duration of response; EFS, event-free survival; ELN, European Leukemia Net; FLT3-ITD, FMS-like tyrosine kinase-3 internal tandem duplication; OS, overall survival;RUNX1;Runt-related transcription factor 1; TET2, ten-eleven translocation 2; U2AF1, U2 small nuclear RNA auxiliary factor 1 gene.

**Supplementary Table 3** Univariate and multivariate analysis of prognostic factors for overall response rate of patients with AML in propensity score matched subgroups

| Variable | Univariate analysis |  | *P* value |  | Multivariate analysis | P value |
| --- | --- | --- | --- | --- | --- | --- |
|  | Response *n* (%) | No response *n* (%) |  |  | OR ( 95% CI ) |  |
| Gender |  |  | 0.974 |  |  |  |
| Male | 29 (70.7) | 12 (29.3) |  |  |  |  |
| Female | 19 (70.4) | 8 (29.6) |  |  |  |  |
| Age (years) |  |  | 0.459 |  |  |  |
| < 40 | 17 (65.4) | 9 (34.6) |  |  |  |  |
| ≥ 40 | 31 (73.8) | 11 (26.2) |  |  |  |  |
| Diagnosis |  |  | 0.016 |  |  | 0.018 |
| AML *de novo* | 45 (76.3) | 9 (23.7) |  |  | 1 |  |
| Secondary AML | 3 (33.3) | 6 (66.7) |  |  | 8.330 (1.429 - 48.554) |  |
| Antecedent allo-HSCT |  |  | 0.349 |  |  |  |
| No | 45 (72.6) | 17 (27.4) |  |  |  |  |
| Yes | 3 (50.0) | 3 (50.0) |  |  |  |  |
| Past HMA exposure |  |  | 0.303 |  |  |  |
| No | 37 (74.0) | 13 (26.0) |  |  |  |  |
| Yes | 11 (61.1) | 7 (38.9) |  |  |  |  |
| Treatment groups |  |  | 0.028 |  |  | 0.058 |
| HMA + CAG | 27 (61.4) | 17 (38.6) |  |  | 1 |  |
| Tislelizumab + HMA + CAG | 21 (87.5) | 3 (12.5) |  |  | 0.239 (0.054 - 1.053) |  |
| Disease status |  |  | 0.763 |  |  |  |
| Refractory | 33 (71.7) | 13 (28.3) |  |  |  |  |
| Relapse | 15 (68.2) | 7 (31.8) |  |  |  |  |
| Bone marrow blast (%) |  |  | 0.741 |  |  |  |
| < 40 | 38 (69.1) | 17 (30.9) |  |  |  |  |
| ≥ 40 | 10 (76.9) | 3 (23.1) |  |  |  |  |
| ELN 2022 risk classification |  |  | 0.075 |  |  | 0.172 |
| Favorable | 15 (88.2) | 2 (11.8) |  |  | 1 |  |
| Intermediate/Adverse | 33 (64.7) | 18 (35.3) |  |  | 3.216 (0.601 - 17.204) |  |
| Normal karyotype |  |  | 0.504 |  |  |  |
| No | 21 (75.0) | 7 (25.0) |  |  |  |  |
| Yes | 27 (67.5) | 13 (32.5) |  |  |  |  |
| DNA methylation-related gene mutations^a^ |  |  | 0.455 |  |  |  |
| No | 29 (67.4) | 14 (32.6) |  |  |  |  |
| Yes | 19 (76.0) | 6 (24.0) |  |  |  |  |
| RAS pathway-related gene mutations^a^ |  |  | 0.727 |  |  |  |
| No | 34 (69.4) | 15 (30.6) |  |  |  |  |
| Yes | 14 (73.7) | 5 (26.3) |  |  |  |  |
| Transcription-related gene mutations^a^ |  |  | 0.147 |  |  |  |
| No | 27 (64.3) | 15 (35.7) |  |  |  |  |
| Yes | 21 (80.8) | 5 (19.2) |  |  |  |  |
| RNA splicing-related gene mutations^a^ |  |  | 1.000 |  |  |  |
| No | 42 (70.0) | 18 (30.0) |  |  |  |  |
| Yes | 6 (75.0) | 2 (25.0) |  |  |  |  |

^a^DNA methylation-related gene mutation include TET2, DNMT3A, IDH1, IDH2, and WT1. RAS pathway-related gene mutations include KRAS, NRAS, FTL3-ITD, PTPN11, KIT, and CBL. Transcription-related gene mutations include RUNX1, BCOR, CEBPA, and NPM1. RNA splicing-related gene mutations include SRSF2 and U2AF1.

allo-HSCT, allogeneic hematopoietic stem cell transplantation; AML, acute myeloid leukemia; ASXL1, additional sex comb like 1; CEBPA-bZip, CCAAT/enhancer-binding protein α- basic leucine zipper; HMA, hypomethylating agent; CAG, decitabine/azacitidine, cytarabine, idarubicin/aclarubicin, G-CSF; ELN, European LeukemiaNet; FLT3-ITD, FMS-like tyrosine kinase-3 internal tandem duplication; RUNX1, runt-related transcription factor 1;TET2, ten-eleven translocation 2; U2AF1,U2 small nuclear RNA auxiliary factor 1.

**Supplementary Table 4** Hematologic and nonhematologic treatment-related toxicities of all cycles in the tislelizumab + HMA + CAG group^*^

| Adverse event | Grade | | | | Total |
| --- | --- | --- | --- | --- | --- |
|  | 4 | 3 | 2 | 1 |  |
| Immune-related adverse events |  |  |  |  |  |
| Immune-related thyroiditis |  | 1(2.3) |  |  | 1(2.3) |
| Infection |  |  |  |  |  |
| Febrile neutropenia |  | 21(48.8) |  |  | 21(48.8) |
| Lung infection | 1(2.3) | 7(16.3) |  |  | 8(18.6) |
| Sepsis |  | 4(9.3) |  |  | 4(9.3) |
| Small intestine infection |  | 2(4.7) |  |  | 2(4.7) |
| Anal mucositis |  | 1(2.3) |  |  | 1(2.3) |
| Hematologic treatment-related toxicities |  |  |  |  |  |
| Neutropenia | 41(95.3) | 2(4.7) |  |  | 43(100) |
| Thrombocytopenia | 41(95.3) | 2(4.7) |  |  | 43(100) |
| Anemia |  | 41(95.3) | 2(4.7) |  | 43(100) |
| Nonhematologic treatment-related toxicities |  |  |  |  |  |
| Blood bilirubin increased |  |  |  | 2(4.7) | 2(4.7) |
| Gamma-glutamyltransferase increased |  |  | 1(2.3) | 1(2.3) | 2(4.7) |
| Hyperglycemia |  |  |  | 1(2.3) | 1(2.3) |
| Hyperphosphatemia |  |  |  | 1(2.3) | 1(2.3) |
| Hypocalcemia |  |  |  | 2(4.7) | 2(4.7) |
| Hypokalemia |  |  |  | 5(11.6) | 5(11.6) |
| Hypophosphatemia |  |  |  | 2(4.7) | 2(4.7) |
| Pericardial effusion |  |  | 1(2.3) |  | 1(2.3) |
| Pleural effusion |  |  |  | 2(4.7) | 2(4.7) |

All data are shown as number of patients (%). ^*^The addition of patients attending each treatment visits (treatment cycles) adds up to 24 + 15 + 4 = 43.

**Supplementary Table 5** Hematologic and nonhematologic treatment-related toxicities of all cycles in historical control group^*^

| Adverse event | Grade | | | | Total |
| --- | --- | --- | --- | --- | --- |
|  | 4 | 3 | 2 | 1 |  |
| Infection |  |  |  |  |  |
| Febrile neutropenia | 24(40) |  |  |  | 24(40) |
| Lung infection |  | 16(26.7) |  |  | 16(26.7) |
| Septicemia |  | 3(5) |  |  | 3(5) |
| Bacteremia |  | 3(5) |  |  | 3(5) |
| Breast infections |  | 1(1.7) |  |  | 1(1.7) |
| Perianal abscess |  | 3(5) |  |  | 3(5) |
| Appendicitis |  |  | 1(1.7) |  | 1(1.7) |
| Gallbladder infection |  |  | 1(1.7) |  | 1(1.7) |
| Hematologic treatment-related toxicities |  |  |  |  |  |
| Neutropenia | 58(96.7) | 2(33.3) |  |  | 60(100) |
| Thrombocytopenia | 58(96.7) | 2(33.3) |  |  | 60(100) |
| Anemia | 5(8.3) | 55(91.7) |  |  | 60(100) |
| Nonhematologic treatment-related toxicities |  |  |  |  |  |
| Alanine aminotransferase increased |  |  | 1(1.7) |  | 1(1.7) |
| Aspartate aminotransferase increased |  |  | 1(1.7) |  | 1(1.7) |
| Hypoalbuminemia |  |  |  | 1(1.7) | 1(1.7) |
| Diarrhea |  | 1(1.7) |  |  | 1(1.7) |
| Oral ulcer |  | 1(1.7) |  |  | 1(1.7) |
| Pleural effusion |  |  |  | 1(1.7) | 1(1.7) |

All data are shown as number of patients (%). ^*^The addition of patients attending each treatment visits (treatment cycles) adds up to 44 + 14 + 2 = 60.

**1.2 Supplementary Figures**

**
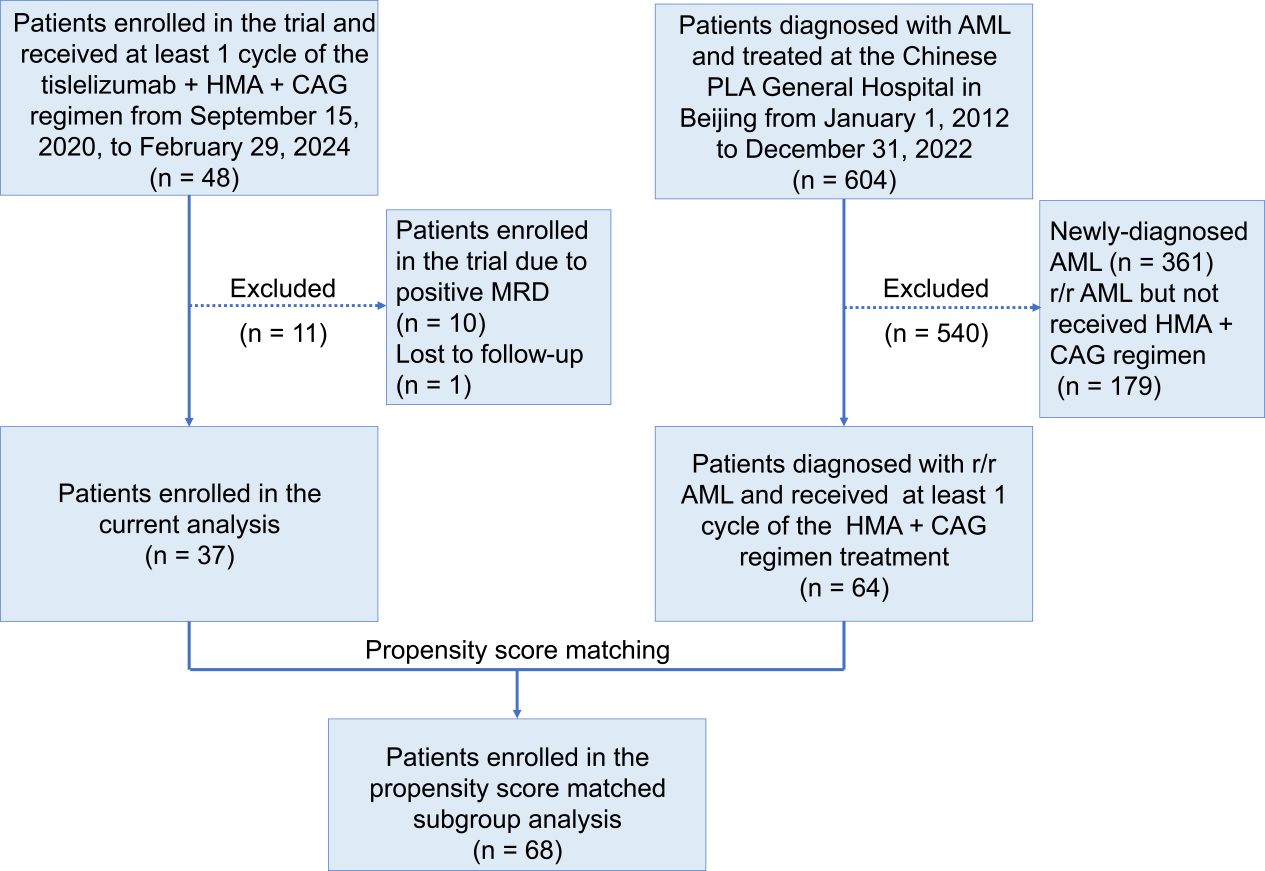
**

**Supplementary Figure 1** The schematic diagram of the clinical study design plan. AML, acute myeloid leukemia; HMA, hypomethylating agent; CAG, decitabine/azacitidine, cytarabine, idarubicin/aclarubicin, G-CSF; MRD, measurable residual disease; r/r, relapsed/refractory.


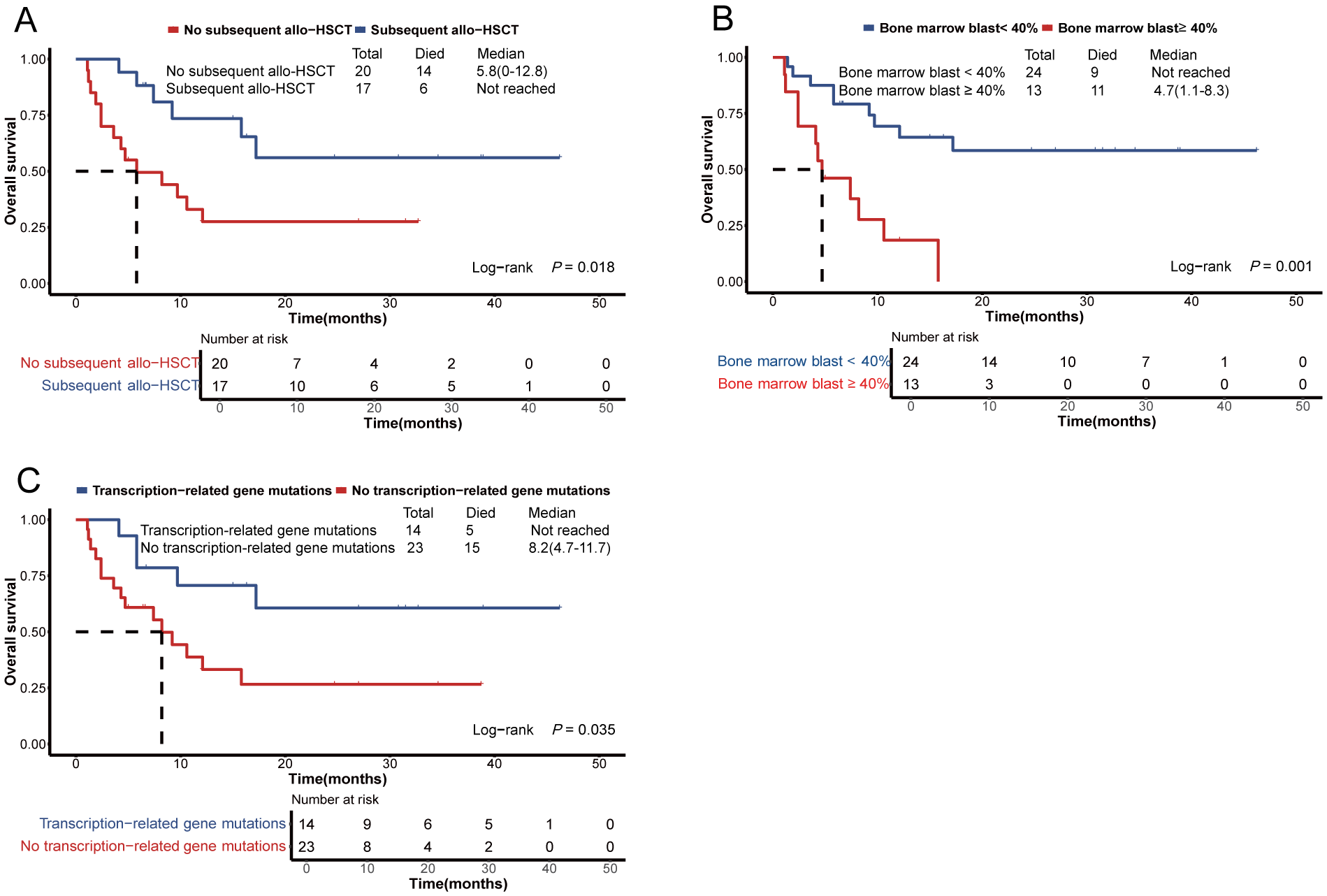


**Supplementary Figure 2** Overall survival plots of the patients with relapsed/refractory AML treated with tislelizumab + HMA + CAG regimen based on subsequent allo-HSCT status **(A)**, bone marrow blast percentage **(B)** or transcription-related gene mutation status **(C).** Allo-HSCT, allogeneic hematopoietic stem cell transplantation; HMA, hypomethylating agent; CAG, decitabine/azacitidine, cytarabine, idarubicin/aclarubicin, G-CSF


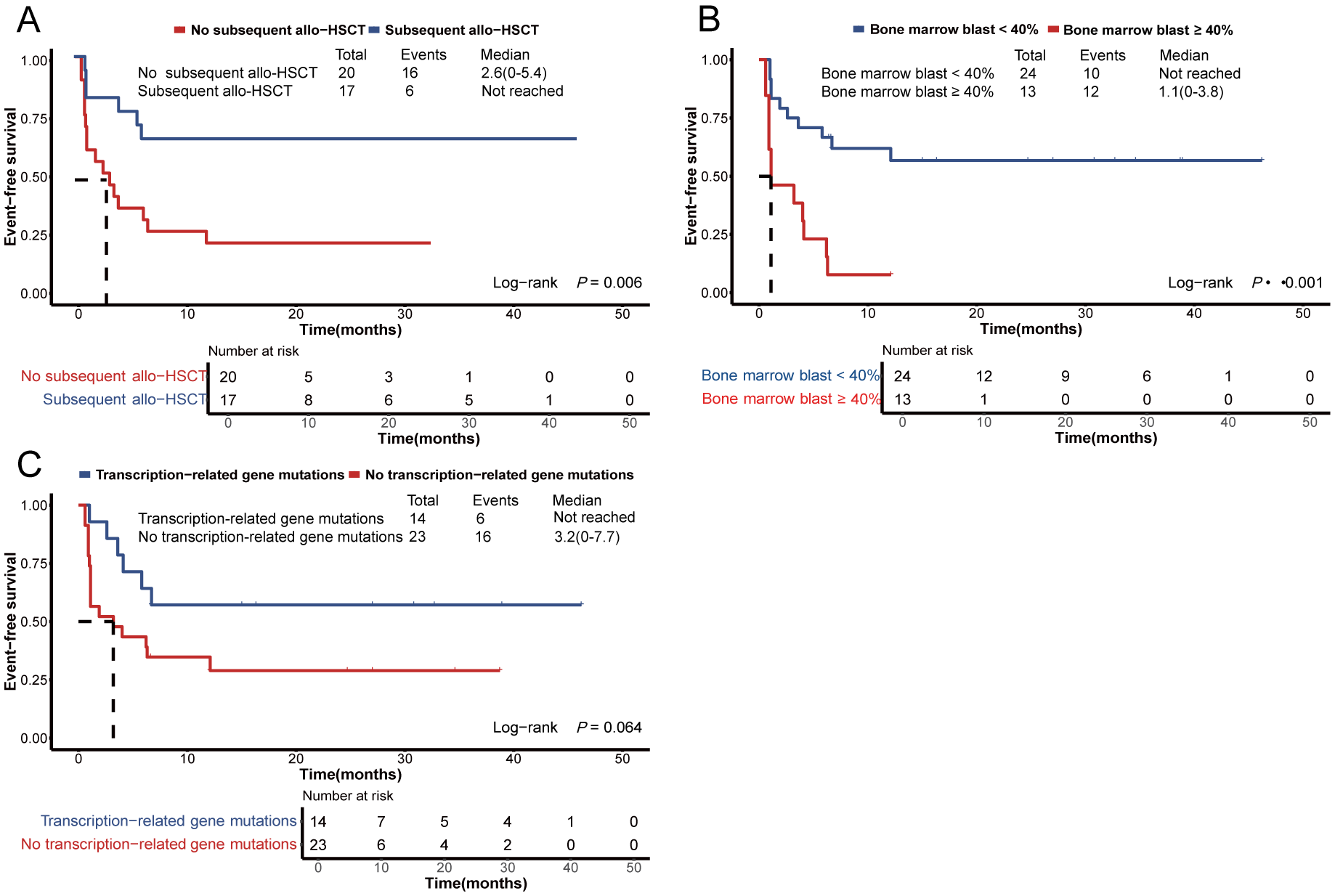


**Supplementary Figure 3** Event-free survival plots of the patients with relapsed/refractory AML treated with tislelizumab + HMA + CAG regimen based on subsequent allo-HSCT status **(A)** or bone marrow blast percentage **(B)** or transcription-related gene mutation status **(C)**. Abbreviations: Allo-HSCT, allogeneic hematopoietic stem cell transplantation; HMA, hypomethylating agent; CAG, decitabine/azacitidine, cytarabine, idarubicin/aclarubicin, G-CSF.

**
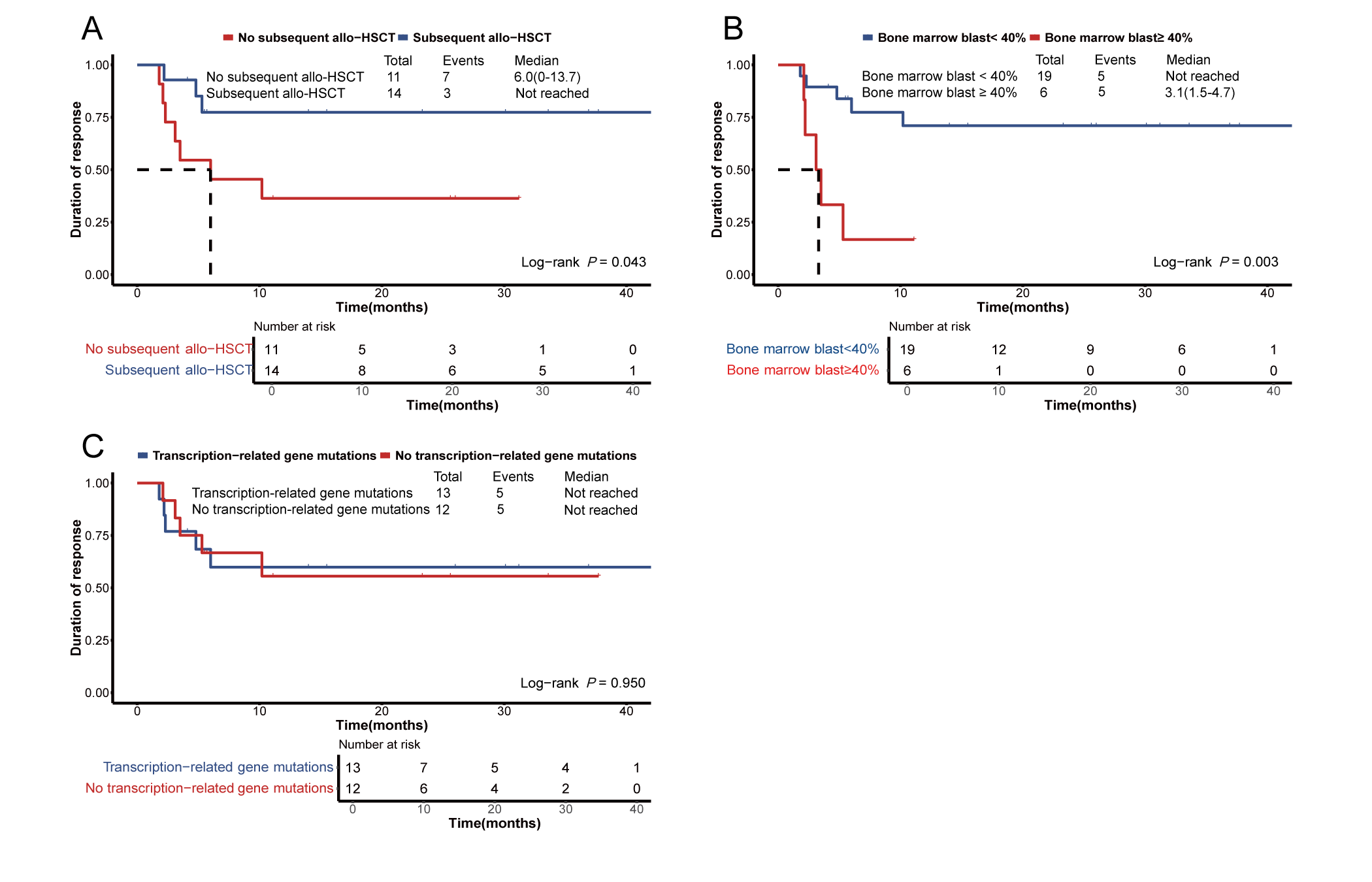
**

**Supplementary Figure 4** Duration of response of the patients with relapsed/refractory AML treated with tislelizumab + HMA + CAG regimen based on subsequent allo-HSCT status **(A)** or bone marrow blast percentage **(B)** or transcription-related gene mutation status **(C)**. Allo-HSCT, allogeneic hematopoietic stem cell transplantation; HMA, hypomethylating agent; CAG, decitabine/azacitidine, cytarabine, idarubicin/aclarubicin, G-CSF.
